# Plot protein: visualization of mutations

**DOI:** 10.1186/2043-9113-3-14

**Published:** 2013-07-22

**Authors:** Tychele Turner

**Affiliations:** 1 Center for Complex Disease Genomics, McKusick-Nathans Institute of Genetic Medicine, Johns Hopkins University School of Medicine, 733 N. Broadway, MRB 572, Baltimore, MD 21205, USA

**Keywords:** Protein, Mutation, Disease, Cluster, Visualization, Plot

## Abstract

**Background:**

Next-generation sequencing has enabled examination of variation at the DNA sequence level and can be further enhanced by evaluation of the variants at the protein level. One powerful method is to visualize these data often revealing patterns not immediately apparent in a text version of the same data. Many investigators are interested in knowing where their amino acid changes reside within a protein. Clustering of variation within a protein versus non-clustering can show interesting aspects of the biological changes happening in disease.

**Finding:**

We describe a freely available tool, Plot Protein, executable from the command line or utilized as a graphical interface through a web browser, to enable visualization of amino acid changes at the protein level. This allows researchers to plot variation from their sequencing studies in a quick and uniform way. The features available include plotting amino acid changes, domains, post-translational modifications, reference sequence, conservation, conservation score, and also zoom capabilities. Herein we provide a case example using this tool to examine the RET protein and we demonstrate how clustering of mutations within the protein in Multiple Endocrine Neoplasia 2A (MEN2A) reveals important information about disease mechanism.

**Conclusions:**

Plot Protein is a useful tool for investigating amino acid changes and their localization within proteins. Command line and web server versions of this software are described that enable users to derive visual knowledge about their mutations.

## Findings

### Background

Researchers are now able to access sequencing datasets across hundreds and thousands of individuals to identify millions of sequence variants [1]. With this onslaught of data comes the task of *understanding* the potential role of these variants for studies of human disease and biology. Many annotation and prediction tools exist for analyzing such variants but visualization of these changes have primarily been restricted to the DNA level, in particular in the UCSC genome browser [2]. We have created a tool (Plot Protein) that can visualize amino acid changes at the protein level identified across individuals. This utility provides users with the capability of observing where their variants lie within the protein and whether the distribution of deleterious mutations is clustered within specific domains. In particular, our tool has the capability of being executed from the command line as R code or from a web browser. The advantage of this implementation is that it gives skilled programmers the opportunity to scale up this analysis to many proteins (command line) or complicated searches across protein families, as well as giving individuals with little to no programming skills the opportunity to view their data interactively in a web browser. Once data is uploaded to the web server the user is then able to quickly tab between the plot and a table displaying annotation of the variation with respect to domains and post-translational modifications. Additionally, in one implementation of the tool (Plot Protein with Conservation) the user has the ability to choose from a number of features including the ability to zoom into any region they would like to and as with the full plot the user merely has to right-click to save as a picture on their computer (for use in reports and publications). Lastly, the benefit of this tool is that it has been written generically to allow users a range of flexibility for the choice of data they wish to include. The user directly provides files on domains and post-translational modifications. This frees the users from databases, which may not contain the exact data they are interested in. Theoretically, amino acid changes from any species can be plotted with this tool making it broadly useful to the scientific community.

## Methods

The command line version of Plot Protein was written in R (http://www.R-project.org) and has been tested on version 2.15.2. It is executed on one line of a terminal window and takes as input a mutation file (protein, gene, position of amino acid change, reference amino acid, and alternate amino acid), a protein architecture file (architecture name, start site, and end site), a post-translational modification file (site position), the length of the protein sequence, and the query name desired on the plot. The alternate implementation of this tool (Plot Protein with Conservation) utilizes the read.fasta function from the seqinr package [3](version 3.0-7) and can also incorporate multiple sequence alignments such as that produced by MUSCLE [4]. To generate a conservation track the multiple sequence alignment is read and all sequences are compared to the user-defined reference at each position. The score (s) is defined simply as s = n/t.

Where n is the number of sequences with the same amino acid at that position as the reference and t is the total number of sequences queried at that position. The score is between 0 and 1 with 0 indicating no other sequences matching the reference at that position and 1 indicating all sequences matching the reference at that position. Additional options include the ability to plot a second set of mutations, show conservation with or without the score, add grid lines, and show the reference sequence. The web version of this tool is comprised of the same R script tools but has been wrapped into a Shiny application using the shiny package (http://www.rstudio.com/shiny/, version 0.4.1.99). This tool requires the same files/data as the command line but with the advantage that files can be easily uploaded/entered via the web. In addition, the web tool also generates a table with the amino acids annotated as to their presence/absence in the domains and post-translational modification sites. This has been extensively tested on Mozilla FireFox and Google Chrome. We also provide the Shiny version of the codes in case users are interested in running this on their own server or locally on their computer.

## Results

**Figure 1:**
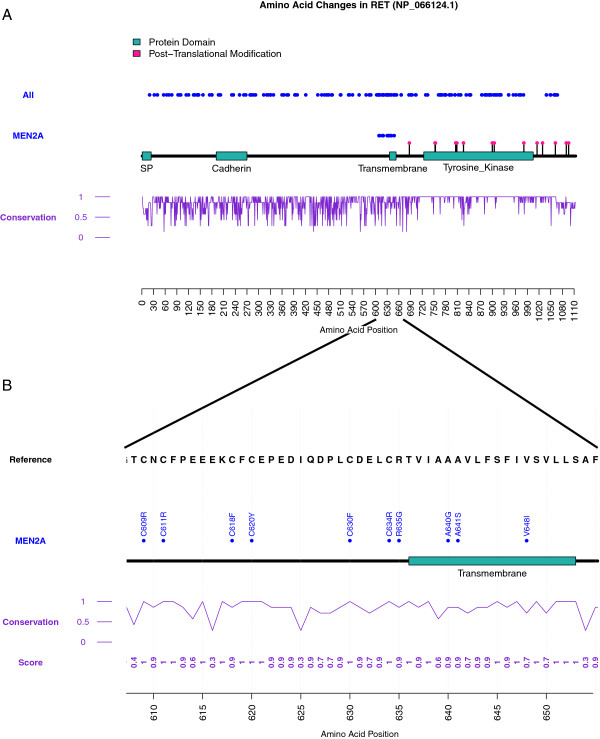
**Output figure of Plot Protein with the conservation track. (A)** Plot of all disease-causing mutations in RET (All track) and those only occurring in MEN2A (MEN2A track) (SP refers to Signal Peptide); **(B)** Zoom in of the region containing the MEN2A mutations including representative labels for each amino acid position.

We have tested our code on numerous datasets but provide an example using amino acid changes within the human ret proto-oncogene (RET) protein. We chose this protein to display all known disease-causing mutations in the gene (n=200) and also a subset of these mutations which are involved in Multiple Endocrine Neoplasia 2A (MEN2A) (n=24) to provide a good example of non-clustered and clustered disease mutation data. All mutations were accessed from the Human Gene Mutation Database (HGMD Professional 2013). These changes map to the NP_066124.1 [RefSeq] isoform of the protein. Domains (Signal Peptide (SP) from 1 to 24, Cadherin from 191 to 270, Transmembrane from 636 to 653, and Tyrosine Kinase from 724 to 1005) and post-translational modifications were derived from the Human Protein Reference Database (HPRD: http://hprd.org/, accessed March 2013) [5]. For the conservation analysis a multiple sequence alignment was generated using the following orthologs of human RET: zebrafish [RefSeq: NP_858048.2], rat [RefSeq: NP_036775.2], mouse [RefSeq: NP_033076.2], gorilla [RefSeq: XP_004049341.1], dog [RefSeq: NP_001184028.1], and cow [RefSeq: NP_001178412.1]. These were run through MUSCLE (http://www.ebi.ac.uk/Tools/msa/muscle/, March 2013) using the default settings with fasta as the output format. The results of this analysis are shown using the conservation implementation in Figure 1. The full protein plot is shown in Figure 1A including a track with all disease mutations and a track with MEN2A only mutations. As can been all disease mutations are distributed fairly evenly across the protein. However, the amino acid changes involved in MEN2A cluster within a particular region of RET (zoom in of the region shown in Figure 1B).

## Discussion

This study describes a tool designed to allow users to review their mutation data, at the protein level, through either the command line or a web server. As an example, we chose to examine disease causing amino acid changes in the RET protein. This case was chosen because of the known clustering of mutations in RET in the MEN2A disease [6,7] which is something that is not seen in all disease mutations affecting RET (Figure 1A). In MEN2A, mutations primarily occur in cysteine residues near the transmembrane domain [6,7]. These mutations cause a dominant gain of function by affecting the folding of the protein and ultimately causing the formation of constitutively active RET dimers (even without ligand) [6,7]. The reason for the clustering only within particular cysteines has been proposed to be because of the proximity to and potential involvement of the transmembrane domain in the stabilization of constitutively active dimers of RET [8]. Since RET is a receptor tyrosine kinase these mutations cause aberrant and uncontrolled signaling ultimately leading to cancer [6,7].

It is likely other diseases also display clustering of mutations due to their effect on function of proteins. Plot Protein could be helpful to researchers in visualizing and identifying these events. Overall, we anticipate that many researchers will find this tool to be useful to their studies regardless of the species they investigate. In addition, we have provided both command line and web versions of this software so that all individuals regardless of their computational prowess can take advantage of this software.

## Availability and requirements

Project name: Plot Protein

Project  home  page: https://sites.google.com/site/plotprotein/

Operating system(s): Mac/Linux for command line

Programming language: R

Other requirements: For running in the web browser best functionality is found with Mozilla Fire Fox and Google Chrome

License: GNU  General  Public  License  version  3.0  (GPLv3), MIT License

Any restrictions to use by non-academics: license needed

## Abbreviations

MEN2A: Multiple Endocrine Neoplasia 2A.

## Competing interests

The author declares they have no competing interests.

## Authors’ contributions

TT designed the software, implemented the software into a freely available web server, analyzed the data, and wrote the publication.
